# Ab-Initio Study of Magnetically Intercalated Platinum Diselenide: The Impact of Platinum Vacancies

**DOI:** 10.3390/ma14154167

**Published:** 2021-07-27

**Authors:** Peter D. Reyntjens, Sabyasachi Tiwari, Maarten L. Van de Put, Bart Sorée, William G. Vandenberghe

**Affiliations:** 1 Department of Materials Science and Engineering, The University of Texas at Dallas, 800 W Campbell Rd, Richardson, TX 75080, USA; peter.reyntjens@utdallas.edu (P.D.R.); sabyasachi.tiwari@utdallas.edu (S.T.); maarten.vandeput@utdallas.edu (M.L.V.d.P.); 2 Department of Materials Engineering, KU Leuven, Kasteelpark Arenberg 44/Box 2450, 3001 Leuven, Belgium; 3 IMEC, Kapeldreef 75, 3001 Leuven, Belgium; bart.soree@imec.be; 4 Department of Electrical Engineering, KU Leuven, Kasteelpark 10, 3001 Leuven, Belgium; 5 Department of Physics, University of Antwerp, Groenenborgerlaan 161, 2020 Antwerp, Belgium

**Keywords:** transition metal dichalcogenides, magnetism, Monte Carlo

## Abstract

We study the magnetic properties of platinum diselenide (PtSe_2_) intercalated with Ti, V, Cr, and Mn, using first-principle density functional theory (DFT) calculations and Monte Carlo (MC) simulations. First, we present the equilibrium position of intercalants in ${\mathrm{PtSe}}_{2}$ obtained from the DFT calculations. Next, we present the magnetic groundstates for each of the intercalants in ${\mathrm{PtSe}}_{2}$ along with their critical temperature. We show that Ti intercalants result in an in-plane AFM and out-of-plane FM groundstate, whereas Mn intercalant results in in-plane FM and out-of-plane AFM. V intercalants result in an FM groundstate both in the in-plane and the out-of-plane direction, whereas Cr results in an AFM groundstate both in the in-plane and the out-of-plane direction. We find a critical temperature of <0.01 K, 111 K, 133 K, and 68 K for Ti, V, Cr, and Mn intercalants at a 7.5% intercalation, respectively. In the presence of Pt vacancies, we obtain critical temperatures of 63 K, 32 K, 221 K, and 45 K for Ti, V, Cr, and Mn-intercalated ${\mathrm{PtSe}}_{2}$, respectively. We show that Pt vacancies can change the magnetic groundstate as well as the critical temperature of intercalated ${\mathrm{PtSe}}_{2}$, suggesting that the magnetic groundstate in intercalated ${\mathrm{PtSe}}_{2}$ can be controlled via defect engineering.

## 1. Introduction

The field of two-dimensional (2D) spintronics [1] has seen unprecedented attention in the past few years, thanks to the recent experimental discovery of two-dimensional (2D) magnetic materials ${\mathrm{CrI}}_{3}$ [2,3] and ${\mathrm{CrGeTe}}_{3}$ [4]. However, the low Curie temperature of experimentally discovered 2D magnets (45 K for monolayer and 61 K for bulk ${\mathrm{CrI}}_{3}$) impedes their technological application. Thankfully, there are many possible 2D magnets. One avenue of finding such 2D magnets is searching the space of magnetic crystals [5,6]. Another avenue of realizing 2D magnets is through magnetic doping [7,8] of conventional 2D materials. The advantage of such magnetically doped magnets is the ability to control their properties through charge transfer.

Transition metal dichalcogenides (TMDs) offer a promising avenue for realizing 2D magnets through magnetic doping [7,8,9,10,11,12,13]. There have been many theoretical [7,8,11,12,14] and experimental [9,10,13] reports of realizing 2D magnetism in semiconducting TMDs through metal doping. On the theoretical side, Mishra et al. [11] investigated the effect of Mn doping on the magnetic properties of MoS_2_, MoSe_2_, MoTe_2_, and WS_2_. Mn-doped MoS_2_ was also studied by A. Ramasubramaniam and D. Naveh [12], who obtained promising results predicting magnetic order persisting above room temperature. The work of Muhammad H. et al. [9] combined experimental fabrication and characterization with DFT methods to investigate the magnetic properties of Ni-doped WSe_2_, which they reported to have room-temperature magnetic ordering. Luo et al. produced a review paper discussing the doping and functionalization of several different 2D TMDs [15], including MoS_2_, MoSe_2_, WS_2_, and WSe_2_. However, similar work on realizing magnetic order in metallic TMDs with the 1T structure is not as common. As recent reports on 2D metallic magnetic systems, e.g., ${\mathrm{Fe}}_{3}{\mathrm{GeTe}}_{2}$ [16], show room-temperature magnetic order, it is natural to look at other metallic systems, such as PtSe_2_.

Metallic TMDs are interesting because free electrons can provide a pathway for long-range magnetic interaction. One such interesting metallic TMD is ${\mathrm{PtSe}}_{2}$. ${\mathrm{PtSe}}_{2}$ forms a layered TMD with semimetallic character [17]. More recently, epitaxial growth of mono- and few-layer ${\mathrm{PtSe}}_{2}$ has revealed a transition to semiconducting character for the monolayer and bilayer ${\mathrm{PtSe}}_{2}$ [18,19]. Moreover, the large atomic weight of ${\mathrm{PtSe}}_{2}$ can result in a higher anisotropy, which is necessary for the existence of 2D magnetic order [20]. Kar et al. performed a first-principles study of the magnetism in doped PtSe_2_ monolayers and found promising higher-than-room-temperature magnetic ordering [21]. However, detailed work on the magnetic ordering, including Monte Carlo simulations for the estimation of the transition temperature in magnetically doped PtSe_2_, is still missing from the literature. The presence of Pt vacancies in pristine ${\mathrm{PtSe}}_{2}$ is responsible for a spin polarization of the electronic cloud around the vacancy, making defective ${\mathrm{PtSe}}_{2}$ a material of interest for future spintronic applications [22,23]. Moreover, theoretical studies have shown that the presence of vacancies in TMDs is energetically favorable in pristine ${\mathrm{PtSe}}_{2}$ grown in Se-rich conditions [24] and reduces the formation energy of intercalated systems [8]. In Ref. [24], the authors find a Pt vacancy density of $4.3\pm 1.4\times 10^{12}/{\mathrm{cm}}^{2}$ in ultrathin layered ${\mathrm{PtSe}}_{2}$.

In this work, we theoretically investigate the magnetic order in bulk ${\mathrm{PtSe}}_{2}$ intercalated with Ti, V, Cr, and Mn, using first-principles density functional theory (DFT) calculations and Monte Carlo (MC) simulations. In Section 2, we describe the methods used in our work, starting from DFT for the calculation of structural parameters and magnetic groundstates, up to the critical temperature calculation using Monte Carlo. In Section 3, we first present the equilibrium position of intercalants in ${\mathrm{PtSe}}_{2}$ obtained from the DFT calculations. Next, we present the magnetic groundstates for each of the intercalants in ${\mathrm{PtSe}}_{2}$ along with their critical temperature. We study the effect of Pt vacancies on the structure and formation energy and investigate their impact on the magnetic order. Finally, in Section 4, we conclude.

## 2. Methods

**Figure 1:**
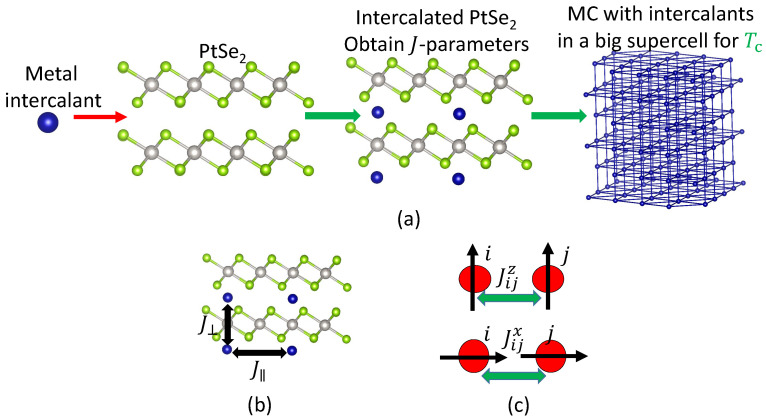
(**a**) The magnetic ions are intercalated in ${\mathrm{PtSe}}_{2}$. (**b**) The exchange parameters in the in-plane ($J_{\|}$) and in the out-of-plane ($J_{⊥}$) direction. (**c**) The *J* parameters between spins *i* and *j* oriented in the in-plane direction ($J_{ij}^{x}$) and in the out-of-plane direction ($J_{ij}^{z}$).

Figure 1a illustrates our computational model. We first intercalate a 2×2×2 supercell of ${\mathrm{PtSe}}_{2}$ and calculate the total energy of various magnetic configurations including ferromagnetic (FM) and anti-ferromagnetic (AFM) configurations using DFT+*U* calculations. Details on the computational parameters are included in Appendix A. To take into account the magnetic anisotropy, we perform total energy calculations with spin-axis oriented in the in-plane and the out-of-plane direction.

Next, we model the magnetic structure of an intercalated ${\mathrm{PtSe}}_{2}$ supercell using a parameterized Heisenberg Hamiltonian:

(1) $$H=\frac{1}{2}\sum_{i,j}S_{i}J_{ij}S_{j}+\sum_{i}D{\left(S_{i}^{z}\right)}^{2},$$

where $S=S^{x}\hat{x}+S^{y}\hat{y}+S^{z}\hat{z}$ is the magnetic moment vector of the intercalant atom. The exchange interaction strength tensor [7], with elements $J_{ij}$ describing the strength of the interaction between spins at site *i* and *j*, is assumed to be rotationally invariant in the direction of the ${\mathrm{PtSe}}_{2}$ planes, because ${\mathrm{PtSe}}_{2}$ has in-plane rotational symmetry. Additionally, we assume that the interaction strength in the *x* and the *y* directions is equal and that the tensor’s diagonal elements are vanishingly small. The result is a diagonal exchange interaction strength tensor with elements $J_{ij}$ where $J_{ij}^{x}=J_{ij}^{y}$. We consider the range of interaction up to the nearest-neighbor atoms in the in-plane and the out-of-plane direction shown in Figure 1b. The second term is the single-ion anisotropy *D*. With $J_{ij}$ being diagonal and rotationally invariant in the plane of the ${\mathrm{PtSe}}_{2}$ layers, the Heisenberg Hamiltonian becomes

(2) $$H=\frac{1}{2}\sum_{i,j}\left(S_{i}^{z}J_{ij}^{z}S_{j}^{z}+S_{i}^{x}J_{ij}^{x}S_{j}^{x}\right)+\sum_{i}D{\left(S_{i}^{z}\right)}^{2},$$

Here, x/z is the direction of spins, when oriented in the in-plane/out-of-plane direction, as shown in Figure 1c. The parameters $J_{ij}^{x/z}$ and *D* are obtained by fitting to the total-energy DFT calculations using the method developed in Ref. [25]. To assess the validity of the nearest-neighbor approximation, we have performed additional calculations to investigate the effect of the second-nearest-neighbor interactions, of which the details are explained in Section S1 of the Supplementary Information.

After obtaining the parameters of the Heisenberg Hamiltonian, we make larger supercells of intercalated ${\mathrm{PtSe}}_{2}$ (8×8×8, or 512 magnetic sites), and study the magnetic phase transition using MC simulations. For each material, we perform ten independent MC runs, each with a different initial condition. We simulate the magnetic order using 3000 equilibration steps and 3000 subsequent MC steps. From the MC simulations, we obtain the specific heat and the magnetization of the intercalated ${\mathrm{PtSe}}_{2}$ as a function of temperature. From the peak of the specific heat, we determine the critical (Curie/Néel) temperature.

## 3. Results and Discussion

### 3.1. Structure of Intercalated PtSe_2_

**Figure 2:**
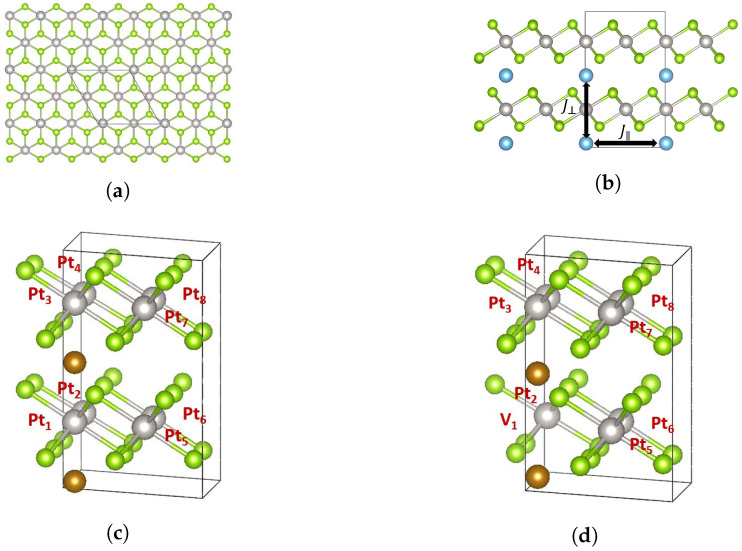
(**a**) Top view of the intercalated ${\mathrm{PtSe}}_{2}$. The original unit cell has 8 Pt atoms, 16 Se atoms, and 2 intercalant atoms. (**b**) The side view of the intercalated ${\mathrm{PtSe}}_{2}$. The in-plane ($J_{\|}$) and out-of-plane ($J_{⊥}$) exchange interactions are shown as well. (**c**) The ${\mathrm{PtSe}}_{2}$ structure without vacancies. (**d**) The ${\mathrm{PtSe}}_{2}$ structure with a Pt vacancy at the location of Pt_1_.

Figure 2 shows the structure of intercalated ${\mathrm{PtSe}}_{2}$. Figure 2a,b show the top and the side view of the most stable structure of the intercalated PtSe_2_ for each of Ti, V, Cr, and Mn intercalation (see Section S2 of the Supplementary Information for structural details, and Section S3 for details on the formation energy calculations), obtained from DFT relaxation in a 2×2×1 supercell.

We define the intercalation fraction according to the number of potential intercalant sites. The intercaltion of 3*d* transition metals into MX_2_ TMDs (with M = metal and X = chalcogen), such as ${\mathrm{PtSe}}_{2}$ and WSe_2_, typically happens at sites with octahedral coordination of X atoms [26]. Therefore, since we have one intercalant for every four octahedral intercalation sites, we express the intercalated ${\mathrm{PtSe}}_{2}$ as TM_1/4_${\mathrm{PtSe}}_{2}$ (where TM = intercalant atom). We use the same DFT-relaxed TM_1/4_${\mathrm{PtSe}}_{2}$ in all subsequent calculations. We choose the fraction TM_1/4_ for our study because the intercalants are likely to form an ordered superlattice along the *c* axis of the hexagonal unit cells (i.e., in the out-of-plane direction) [26,27].

Figure 2c,d compare the ${\mathrm{PtSe}}_{2}$ structure with and without the presence of the vacancy V_1_ at the site of atom Pt_1_. We find that the Pt vacancy causes the structure to change in the out-of-plane direction; see Table S1.

### 3.2. Magnetic Ordering and Critical Temperature

**Figure 3:**
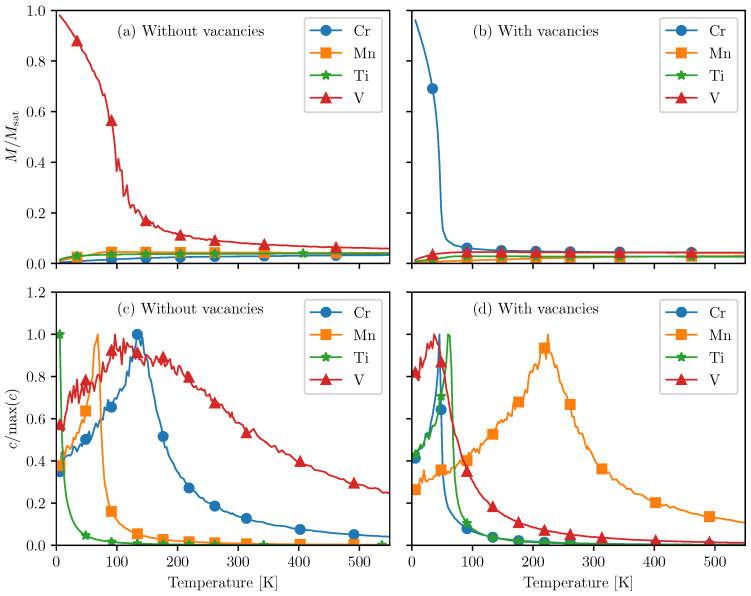
(**a**) The magnetization of intercalated ${\mathrm{PtSe}}_{2}$ for various intercalants without any vacancies. *M* is the magnetization per intercalant atom obtained from the MC simulations and $M_{\mathrm{sat}}$ is the saturation magnetization. (**b**) The magnetization of intercalated ${\mathrm{PtSe}}_{2}$ for various intercalants in the presence of vacancies. (**c**) The specific heat *c* of intercalated ${\mathrm{PtSe}}_{2}$ for various intercalants without any vacancies. (**d**) The specific heat *c* of intercalated ${\mathrm{PtSe}}_{2}$ in the case where vacancies are present.

Figure 3a shows the magnetization as a function of temperature for intercalated PtSe_2_ for intercalants Ti, V, Cr, and Mn without Pt vacancies. We normalize the curves to the saturation magnetization $M_{\mathrm{sat}}$, which is the maximum magnetization that can be achieved in the material, when all magnetic moments point in exactly the same direction. We observe that when no vacancies are present, the magnetization for V saturates at low temperatures, suggesting a ferromagnetic transition. For Ti, Cr, and Mn, the magnetization vanishes at lower temperatures, suggesting an anti-ferromagnetic transition.

Figure 3b shows the magnetization as a function of temperature for intercalated PtSe_2_ for intercalants Ti, V, Cr, and Mn with Pt vacancies. We observe that when the vacancies are present, the magnetization of Ti-, V-, and Mn-intercalated ${\mathrm{PtSe}}_{2}$ goes to zero, suggesting an anti-ferromagnetic order. For Cr-intercalated ${\mathrm{PtSe}}_{2}$, the magnetization reaches saturation, suggesting a ferromagnetic transition.

Figure 3c shows the specific heat as a function of temperature for intercalated pristine ${\mathrm{PtSe}}_{2}$. We observe that the specific heat peaks at 111K for V, 133K for Cr, and at 68K for Mn. However, for Ti, the specific heat peaks at a much lower temperature, namely below the lowest temperature point in our simulation, 0.01K.

Figure 3d shows the specific heat as a function of temperature for intercalated pristine ${\mathrm{PtSe}}_{2}$. When vacancies are present, the specific heat peaks at 63K, 32K, 221K, and 45K for Ti, V, Cr, and Mn, respectively.

### 3.3. Exchange Interactions and Magnetic Groundstate

Table 1 shows the obtained *J* parameters, onsite anisotropy (*D*), and the magnetic moment ($M_{\mathrm{sat}}$) for various intercalants obtained from the DFT calculations, with and without Pt vacancies. The *J* parameters increase quite significantly for Ti and Mn, boosting their Néel temperature from below 0.01K and 68K in pristine ${\mathrm{PtSe}}_{2}$ to 68K and 221K in defective ${\mathrm{PtSe}}_{2}$, respectively. Moreover, we see that although the out-of-plane *J* parameters ($J_{⊥}^{x/z}$) of V remain positive, the in-plane *J* parameters ($J_{∥}^{x/z}$) become negative, resulting in an in-plane AFM and out-of-plane FM groundstate. The V-intercalated ${\mathrm{PtSe}}_{2}$ therefore changes from a ferromagnet with $T_{C}$ of 111K, to an anti-ferromagnet with a $T_{N}$ of 32K. For Cr, all *J* parameters, both in-plane and out-of-plane, change sign, resulting in a change in the magnetic groundstate from purely anti-ferromagnetic to purely ferromagnetic, when vacancies are present. Cr-intercalated ${\mathrm{PtSe}}_{2}$ changes from an anti-ferromagnet with $T_{N}=133\;K$ when the ${\mathrm{PtSe}}_{2}$ is pristine to a ferromagnet with $T_{C}=45\;K$ when the material contains vacancies. Additionally, we find that for all the intercalants except for Ti, the out-of-plane interaction ($J_{⊥}^{x/z}$) is stronger than the in-plane interaction, suggesting a strong out-of-plane super super-exchange interaction [28].

The interaction between magnetic intercalant atoms occurs through a super super-exchange interaction chain X – Se – Pt – Se – X, where X denotes the intercalant atom (in our case: Ti, V, Cr, or Mn). The d orbitals of the intercalant atoms couple to each other through the p orbitals of the Se atoms and the d orbitals of the Pt atom in the chain. In the super super-exchange mechanism, each step in the chain consists of an anti-ferromagnetic coupling, so that the X atoms are ferromagnetically coupled. In the case where the X atoms have a positive spin density, the chain consists of the following spin density signs: X (+) – Se (−) – Pt (+) – Se (−) – X (+), where “+” refers to a positive spin density, and “−” refers to a negative spin density. When such a ferromagnetic coupling is satisfied, the super super-exchange interactions will stabilize the ferromagnetic state and be the reason that the out-of-plane exchange parameters are large.

In the case of V-intercalated ${\mathrm{PtSe}}_{2}$ without vacancies, we see that the V (+) – Se (−) – Pt (+) – Se (−) – V (+) chain is satisfied; see Figure S3a in the Supplementary Information. From Table 1, we see that the out-of-plane exchange coupling is indeed strong, $J_{⊥}^{z}=3.07\;\mathrm{meV}/\mu_{B}^{2}$ and $J_{⊥}^{x}=3.25\;\mathrm{meV}/\mu_{B}^{2}$ (see Table 1), when compared to the in-plane exchange interaction. Such a coupling through the super super-exchange mechanism stabilizes the ferromagnetic state, which is why the V-intercalated ${\mathrm{PtSe}}_{2}$ without vacancies has a ferromagnetic groundstate.

For Mn-intercalated ${\mathrm{PtSe}}_{2}$, however, we see that the chain has a different form: the Mn (+) – Se (−) – Pt (−) – Se (−) – Mn (+); see Figure S3b in the Supplementary Information. We see that the super super-exchange interaction does not take place, and that because of the lack of super super-exchange interaction, the out-of-plane exchange parameters are small, namely $J_{⊥}^{z}=-0.09\;\mathrm{meV}/\mu_{B}^{2}$ and $J_{⊥}^{x}=0.07\;\mathrm{meV}/\mu_{B}^{2}$; see Table 1. The lack of stabilizing super super-exchange interactions causes an energy penalty in the ferromagnetic state, which pushes the state up in energy. The result is that the groundstate of Mn-intercalated ${\mathrm{PtSe}}_{2}$ is not the ferromagnetic state, but an anti-ferromagnetic state.

Additionally, looking at the effect of vacancies, we can attribute the destabilization of the ferromagnetic state of V-intercalated ${\mathrm{PtSe}}_{2}$ to a disruption of the super super-exchange interactions, causing a drop in the strength of the out-of-plane exchange parameters, namely $J_{⊥}^{z}$ drops from $3.07\;\mathrm{meV}/\mu_{B}^{2}$ to $0.71\;\mathrm{meV}/\mu_{B}^{2}$ and $J_{⊥}^{x}$ drops from $3.25\;\mathrm{meV}/\mu_{B}^{2}$ to $0.72\;\mathrm{meV}/\mu_{B}^{2}$ when the Pt vacancies are considered. The disruption of the super super-exchange mechanism causes the ferromagnetic state to shift up in total energy, and the groundstate goes from being a ferromagnetic state with a Curie temperature of 111K to being an anti-ferromagnetic state with a Néel temperature of 32K.

Comparing the *J* parameters in Table 1, we see that the sign of either the in-plane ($J_{∥}^{x/z}$) or the out-of-plane ($J_{⊥}^{x/z}$) exchange parameters change for all the intercalants. The change in the sign of *J* parameters suggests that the magnetic groundstate changes for all of the intercalated ${\mathrm{PtSe}}_{2}$ with Pt vacancy compared to intercalated pristine ${\mathrm{PtSe}}_{2}$. The tunability of *J* parameters with vacancy suggests that the magnetic properties of intercalated ${\mathrm{PtSe}}_{2}$ can be tuned by creating vacancies in ${\mathrm{PtSe}}_{2}$.

From the DFT results, we extract the localized magnetic moments on each atom of our intercalated materials. We find that the magnetic moments on the Pt and Se atoms are small compared to the moments on the intercalant atoms. The largest relative magnetic moment on a Pt atom occurs in Ti-intercalated ${\mathrm{PtSe}}_{2}$ in the presence of vacancies and its size is 8.43% of that of the Ti atoms. The smallest relative magnetic moment on a Pt atom occurs in pristine Mn-intercalated ${\mathrm{PtSe}}_{2}$ and its size is only 0.5% of the magnetic moment of the Mn atoms. Therefore, we conclude that our assumption that the magnetic moments are mostly located on the intercalant atoms is correct.

## 4. Conclusions

We have theoretically studied the possibility of realizing magnetic order in 1T-${\mathrm{PtSe}}_{2}$ through magnetic intercalation with Ti, V, Cr, and Mn. We showed that Ti results in an in-plane AFM and out-of-plane FM groundstate, whereas Mn results in in-plane FM and out-of-plane AFM. V results in an FM groundstate both in-plane and out-of-plane, whereas Cr results in an AFM groundstate both in the in-plane and out-of-plane direction. The critical temperatures that we find are lower than 0.01K, 111K, 133K, and 68K for Ti, V, Cr, and Mn, respectively.

We have further shown that the Pt vacancy significantly impacts the magnetic order in intercalated ${\mathrm{PtSe}}_{2}$ both qualitatively and quantitatively. Most significantly, V intercalants become in-plane AFM from in-plane FM, and Cr intercalants transition from an AFM groundstate to an FM groundstate. Moreover, the Néel temperature of both Ti and Mn intercalants increases with Pt vacancy to 63K and 221K compared to a $T_{N}$ lower than 0.01K and $T_{N}=68\;K$ in the pristine ${\mathrm{PtSe}}_{2}$, respectively. Finally, we have shown that Pt vacancies can reduce the energy of formation in intercalated ${\mathrm{PtSe}}_{2}$.

The tunability of the magnetic groundstate and critical temperature opens a plethora of opportunities for defect engineering the magnetic groundstate in ${\mathrm{PtSe}}_{2}$ through intercalation. Further exploration of the electronic properties of intercalated ${\mathrm{PtSe}}_{2}$ would provide deeper insights into the tuning of the magnetic order in ${\mathrm{PtSe}}_{2}$. Additionally, we would like to mention that investigating the stability of magnetic states for different intercalant fractions would be an interesting avenue for future studies.

## Figures and Tables

**Table 1:** Summary of the results for the intercalated PtSe_2_ with and without vacancies. Results for structures with vacancies are in the columns marked with (Pt_V_).

Intercalant	Ti		V		Cr		Mn	
Pt Vacancy Yes/No	No	Yes	No	Yes	No	Yes	No	Yes
Formation energy (eV)	−0.39	−2.09	0.77	−0.81	1.44	0.12	1.27	−0.06
*U*-value (eV)	4.23	3.86	4.45	4.01	5.03	3.72	6.80	5.18
Magnetic moment ($\mu_{B}$)	1.52	0.60	2.77	2.6	3.90	3.55	4.44	4.26
$J_{⊥}^{z}$ (meV/$\mu_{B}^{2}$)	0.05	−3.91	3.07	0.71	−0.65	0.04	−0.09	−0.82
$J_{∥}^{z}$ (meV/$\mu_{B}^{2}$)	−0.26	2.79	0.07	−0.09	−0.20	0.25	0.07	−0.25
$J_{⊥}^{x}$ (meV/$\mu_{B}^{2}$)	0.02	−5.16	3.25	0.72	−0.54	0.02	−0.10	−0.75
$J_{∥}^{x}$ (meV/$\mu_{B}^{2}$)	−0.28	3.33	0.06	−0.10	−0.16	0.24	0.07	−0.20
*D* (meV/$\mu_{B}^{2}$)	−0.04	2.31	0.33	0.04	0.38	−0.02	−0.02	0.36
$T_{C}$ (K)	**—**	**—**	111	**—**	**—**	45	**—**	**—**
$T_{N}$ (K)	<0.01	63	**—**	32	133	**—**	68	221

## Data Availability

The data presented in this study are available within the article (and its supplementary material).

## Supplementary Materials

The following are available online at https://www.mdpi.com/article/10.3390/ma14154167/s1, Figure S1: The supercell used in the next-nearest neighbor calculations, Table S1: The lattice vectors and angles for the intercalated PtSe2 structures with and without Pt vacancies, Figure S2: The formation energy of the different intercalated PtSe2 structures, with and without vacancies present, Figure S3: (a) Spin density for V-intercalated PtSe2. (b) Spin density for Mn-intercalated PtSe2.

## Abbreviations

The following abbreviations are used in this manuscript:

TMD	Transition Metal Dichalcogenide
DFT	Density Functional Theory
MC	Monte Carlo
FM	Ferromagnetic
AFM	Anti-ferromagnetic

## Appendix A. DFT Calculations

For all our DFT calculations, we use the Vienna Ab-initio Simulation Package (VASP) [29,30,31,32]. We use the Projector-Augmented Wave (PAW) method [33] and the generalized gradient approximation proposed by Perdew, Burke and Ernzerhof [34] for the exchange and correlation functionals in our calculations. To account for the Van der Waals interactions, we use the DFT-D3 method of Grimme et al. [35]. In all our DFT calculations, we set the plane-wave cutoff energy at 500eV and employ an energetic convergence criterion of $10^{-5}\;\mathrm{eV}$ to ensure our results are accurate. Prior to any self-consistent or Monte Carlo calculation, we perform the structural optimization of our systems by changing the lattice parameters and ionic positions until all the forces on the ions are lower than 0.005eV/Å.

### Appendix A.1. Hubbard U Correction

The intercalants are elements with highly correlated electrons in the 3d-shell. Because we use atoms which have an incomplete 3d-shell as intercalants, we need to consider the correlation effects of electrons in the 3d-shell. We use the Hubbard *U* correction within DFT (DFT + *U*) [36] We estimate the value of the *U* parameter using the linear response method of M. Coccocioni and S. De Gironcoli [37]. For each ${\mathrm{PtSe}}_{2}$ + intercalant system, we use the value of *U* obtained using the linear response method in all spin-polarized noncollinear DFT + *U* calculations for that system. The results are listed in Table 1.

### Appendix A.2. Total Energies of the Magnetic States

The strength of magnetic interactions between the dopants depends on the relative stability of the various magnetic states. To get an accurate picture of the magnetic behavior around the critical (Curie or Néel) temperature, we therefore calculate the total energies of different ferromagnetic, ferrimagnetic and anti-ferromagnetic states for each material. On each intercalant site, we take all ferromagnetic, ferrimagnetic and anti-ferromagnetic combinations of the up and down spins states. Once the different spin configurations have been determined we perform non-collinear DFT + *U* by aligning each configuration along the [100] and [001] directions. We set the size of the initial magnetic moments in our DFT simulations according to the number of unpaired electrons in the d-shell of the intercalant atoms. The final groundstate magnetic moments are lower than the number of unpaired electrons due to the formation of bonds.
