# Supplementary material for: Ab-Initio Study of Magnetically Intercalated Platinum Diselenide: The Impact of Platinum Vacancies
Source: Materials (Basel). 2021 Jul 27;14(15):4167. doi: 10.3390/ma14154167 (PMC8348902; doi:10.3390/ma14154167)
Supplement: Supplementary file 1 [file materials-14-04167-s001.zip › materials-1259932-supplementary.pdf]

materials

Supplementary Materials

Ab-Initio Study of Magnetically Intercalated Platinum Diselenide: The Impact of Platinum Vacancies

Peter D. Reyntjens [1,2,3], Sabyasachi Tiwari [1,2,3], Maarten L. Van de Put [1], Bart Sor'ee [3,4,5] and William G. Vandenberghe [1,*]

1 Department of Materials Science and Engineering, The University of Texas at Dallas, 800 W Campbell Rd,75080, Richardson, TX, USA; peter.reyntjens@utdallas.edu (P.D.R.); sabyasachi.tiwari@utdallas.edu (S.T.); maarten.vandeput@utdallas.edu (M.L.V.d.P.)
2 Department of Materials Engineering, KU Leuven, Kasteelpark Arenberg 44/box 2450, 3001, Leuven, Belgium
3 IMEC, Kapeldreef 75, 3001, Leuven, Belgium; bart.soree@imec.be
4 Department of Electrical Engineering, KU Leuven, Kasteelpark 10, 3001, Leuven, Belgium
5 Department of Physics, University of Antwerp, Groenenborgerlaan 161, 2020, Antwerp, Belgium
* Correspondence: william.vandenberghe@utdallas.edu; Tel.: +1 972-883-5793

1. Effect of the second-nearest neighbor interactions

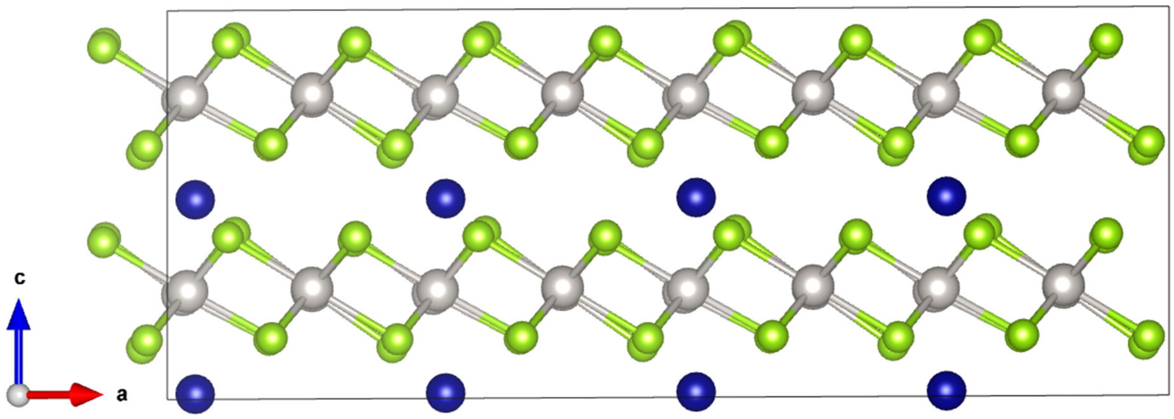

Figure S1. The supercell used in the next-nearest neighbor calculations.

In our model, we have used nearest-neighbor interactions to model the magnetic behavior of the materials. However, one could also include interactions that are further away.

To assess the validity of our model, which includes only nearest-neighbor interactions, we perform additional calculations for $Cr_{1/4}PtSe_2$ (no vacancies), to investigate the strength of second-nearest neighbor in-plane interactions. We find that the second-nearest neighbor in-plane exchange parameters are $J^x_{2NN,\parallel} = -0.027\ \mathrm{meV}/\ \mu_B^2$ and $J^z_{2NN,\parallel} = -0.013\ \mathrm{meV}/\ \mu_B^2$. The nearest neighbor in-plane exchange parameters are $J^x_\parallel = -0.16\ \mathrm{meV}/\ \mu_B^2$ and $J^z_\parallel = -0.20\ \mathrm{meV}/\ \mu_B^2$, as listed in Table 1. Thus, the second-nearest neighbor in-plane interactions are around 1 order of magnitude weaker than the nearest-neighbor interactions, and we can safely ignore them in our model.

2. Lattice structure and parameters

The intercalation of Ti, V, Cr and Mn between the layers of $PtSe_2$ influences the lattice parameters. Additionally, the presence or absence of a Pt vacancy will also affect the lattice parameters. In Table S1, we report the lattice parameters for all our materials.

Table S1. The lattice vectors and angles for the intercalated $PtSe_2$ structures with and without Pt vacancies.

Intercalant	Ti		V		Cr		Mn	
Pt vacancy Yes/No	No	Yes	No	Yes	No	Yes	No	Yes
a	7.50	7.63	7.47	7.57	7.47	7.50	7.45	7.53
b	7.50	7.63	7.47	7.57	7.47	7.50	7.67	7.53
c	11.01	9.81	10.85	9.80	10.48	9.81	10.09	9.80
α	90°	90°	90°	90°	90°	90°	90°	90°
β	90°	90°	90°	90°	90°	90°	90°	90°
γ	120°	120°	120°	120°	120°	120°	119°	120°

Table S1 shows the effect of the intercalants and vacancies on the lattice parameters. The vacancies mainly affect the *c* lattice constant of the various structures. In all cases, *c* is reduced, with the reduction ranging from 2.9% for Mn-intercalated $PtSe_2$ to 10.9% for Ti-intercalated $PtSe_2$. From the angles between the lattice vectors α, β and γ, we conclude that the vacancies do not change the shape of the cell, except for the reduction in the *c* lattice constant.

3. Formation energy

We calculate the formation energy of our materials using,

$$E_{\text{form}} = \frac{1}{M}\left(E_{\text{compound}} - ME_{\text{pure}} - NE_{\text{intercal}}\right) \quad (1)$$

where E_{compound} is the total energy of the intercalated $PtSe_2$, which we obtain from DFT. E_{intercal} is the total energy per atom of the intercalant in its pure, metallic form and E_{pure} is the total energy of the pure $PtSe_2$ unit cell, without any intercalants present. The factor M accounts for the number of unit cell repetitions in the supercell used in the intercalated structures. Finally, N represents the number of intercalant atoms in the supercell.

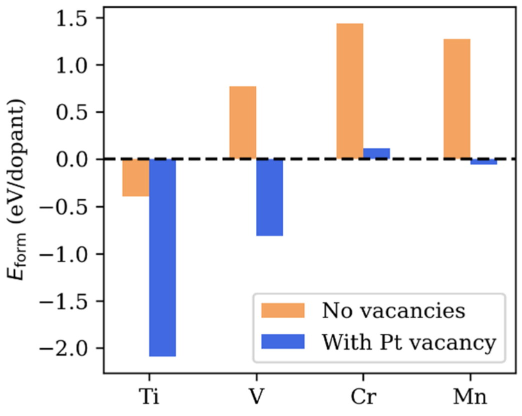

Figure S2. The formation energy of the different intercalated $PtSe_2$ structures, with and without vacancies present.

In Fig. S1, we plot the formation energies of the intercalated $PtSe_2$ with and without the presence of a Pt vacancy in the unit cell. For both cases, the formation energy is lowest for Ti intercalation. Moving from Ti to Mn along the periodic table, the formation energy of the intercalated $PtSe_2$ rises to a maximum for Cr intercalation, before lowering for Mn intercalation.

Strikingly, the addition of Pt vacancies in the material lowers the formation energies by at least 1.32 eV for all our intercalated $PtSe_2$ systems. The largest drop in the formation energy (1.70 eV) happens in Ti-intercalated $PtSe_2$, while the smallest drop (1.32 eV) hap-

pens in Cr-intercalated. A lower formation energy signifies a higher thermodynamic stability of the intercalated $PtSe_2$. Therefore, we can infer that the Pt vacancies significantly impact the stability of the intercalants in $PtSe_2$.

4. Nature of the magnetic interactions: super super-exchange

Here, we present the spin density of V- and Mn-intercalated $PtSe_2$ to provide a visual representation of the super super-exchange interactions in our materials. As mentioned in the main text of our paper, the super super-exchange mechanism connects two intercalant atoms by a chain of TM – Se – Pt – Se – TM atoms, where TM stands for the Transition Metal intercalant atoms. The super super-exchange interaction is ferromagnetic, with an antiferromagnetic coupling at each step in the chain. For example, if the TM atoms are ferromagnetically coupled and have a positive spin density associated with them, the chain is structured as follows: TM (+) – Se (-) - Pt (+) – Se (-) – TM (+), where (+) means that the spin density around the atom is mostly positive.

Figure S3 (a) shows the spin density of the ferromagnetic state of V-intercalated $PtSe_2$, with positive spin density represented by the yellow regions and negative spin density represented by the blue regions. We observe antiferromagnetic coupling between the V atoms and the neighboring Se atoms. Additionally, we observe that the Pt atoms and V atoms both have positive spin density. The super super-exchange coupling is achieved in the V (+) – Se (-) – Pt (+) – Se (-) – V (+) chain, and the out-of-plane exchange constants are ferromagnetic and large, $J_\perp^z = 3.07\ \mathrm{meV}/\mu_\mathrm{B}^2$ and $J_\perp^x = 3.25\ \mathrm{meV}/\mu_\mathrm{B}^2$ (see Table 1). Therefore, the ferromagnetic state is the most stable state in V-intercalated $PtSe_2$.

Fig. S3 (b) shows the spin density of the ferromagnetic state Mn-intercalated $PtSe_2$. We see that here, the Pt atoms are antiferromagnetically coupled to the Mn (intercalant) atoms, and we can represent the chain as Mn (+) – Se (-) – Pt (-) – Se (-) – Mn (+). From the discussion above, we understand that the antiferromagnetic coupling is not favored and disrupts the super super-exchange interactions and destabilizes the ferromagnetic state of Mn-intercalated $PtSe_2$. The lack of super super-exchange interaction can also be seen in the out-of-plane exchange constants, which are relatively low: $J_\perp^z = -0.09\ \mathrm{meV}/\mu_\mathrm{B}^2$ and $J_\perp^x = 0.07\ \mathrm{meV}/\mu_\mathrm{B}^2$, see Table 1. The result is an energy penalty for the ferromagnetic state, and the ground state is antiferromagnetic in Mn-intercalated $PtSe_2$.

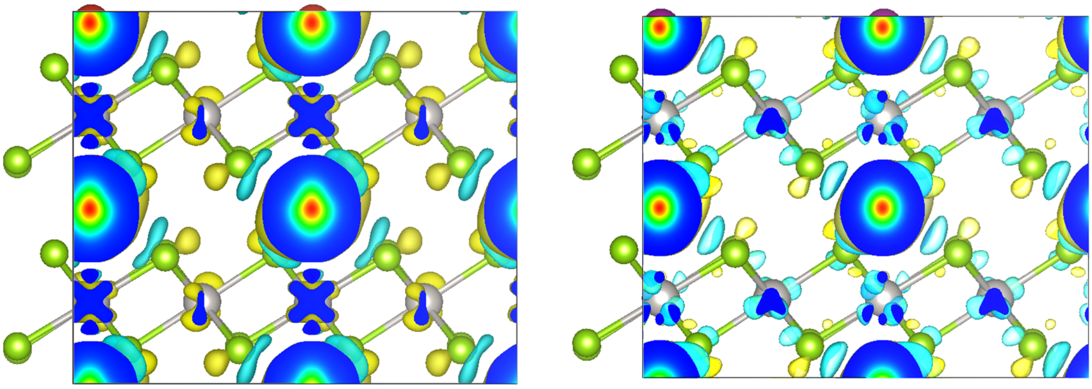

(**a**) (**b**)

Figure S3. (**a**) Spin density for V-intercalated $PtSe_2$. (**b**) Spin density for Mn-intercalated $PtSe_2$.
